# Generalised Versus Specific Internet Use-Related Addiction Problems: A Mixed Methods Study on Internet, Gaming, and Social Networking Behaviours

**DOI:** 10.3390/ijerph15122913

**Published:** 2018-12-19

**Authors:** Olatz Lopez-Fernandez

**Affiliations:** 1 Turning Point, Eastern Health Clinical School, Monash University, 110 Church Street, Richmond VIC 2131, Australia; olatz.lopez-fernandez@monash.edu or lopez.olatz@gmail.com; Tel.: +61-8413-8509; 2 International Gaming Research Unit, Psychology Department, Nottingham Trent University, Nottingham NG1 4FQ, UK; 3 Laboratory for Experimental Psychopathology, Psychological Sciences Research Institute, Catholic University of Louvain, 1348 Louvain-la-Neuve, Belgium

**Keywords:** behavioural addictions, generalised versus specific problem Internet uses, Internet addiction, gaming disorder, social networking, mixed methods research

## Abstract

The field of technological behavioural addictions is moving towards specific problems (i.e., gaming disorder). However, more evidence of generalised versus specific Internet use-related addiction problems (generalised pathological Internet use (GPIU) vs. specific pathological Internet use (SPIU)) is still needed. This mixed methods study aimed to disentangle GPIU from SPIU. A partially mixed sequential equal status study design (QUAN→QUAL) was undertaken. First, through an online survey, which adapted the compulsive Internet use scale (CIUS) for three types of problems (i.e., generalised Internet use, and specific online gaming and social networking). Second, potential problem users’ perceptions of the evolution of these problems (aetiology, development, consequences, and factors) were ascertained, through semi-structured interviews, together with their opinion on present Internet gaming disorder (IGD) criteria adapted to each problem studied. Findings showed the CIUS remains valid and reliable for GPIU and SPIUs examined; a prevalence between 10.8% and 37.4% was estimated for potential at-risk problem gamers and Internet users, respectively, who reported their preference for maintaining their virtual lives. Half of the sample had a risk of a unique or mixed profile of these problems. Moreover, device patterns, gender, and age issues emerged, such as problem gamers being proportionally equal male and female young or middle-aged adults. GPIU was highly associated with problem social networking use, and weakly with problematic gaming, but both SPIUs were independent. Concerning addictive symptoms, salience, deception, and tolerance required redefinition, especially for SPIUs, while better-valued IGD criteria applied to GPIU and SPIUs were: Risk relationships or opportunities, give up other activities, withdrawal, and continue despite problems. Thus, although problems studied are present as risk behaviours, SPIUs seem to cover the addictive symptomatology in those categorised as potential problem users, online gaming being the most severe behavioural addiction problem.

## 1. Introduction

The field of behavioural addictions related to technological uses (i.e., technological behavioural addictions) has been growing exponentially since 1995 [1,2,3,4] and not without scientific, clinical, and social debates. In mid-nineties, the phenomenon was recognised by the umbrella term of ‘Internet addiction’, a generalised addiction problem covering all online activities together. Almost automatically, this was conceptualised as a clinical disorder [5], initially closely aligned with ‘impulse control disorder’. In 2013, it was proposed as a future ‘addictive disorder’ in the third appendix of the fifth Diagnostic and Statistical Manual of Mental Disorders (DSM-5) by the American Psychiatric Association (APA) [6], and at present it has been recognised as a health disease in the eleventh revision of the International Classification of Diseases (ICD-11) by the World Health Organization (WHO) [7]. However, this international recognition has come about solely for a specific technological addictive problem—problematic gaming—even though other technological use-related addiction problems coexist (e.g., cybersex addiction).

The terminology requires an update, as it covers the emergent health issues related to excessive online uses, which emerged together with the development of technologies at the end of last century, and it has been consolidated in the 21st Century. Thus, although it seems Internet use-related addiction problems were initially mainly studied as a generalised problem [8], there is a scientific and clinical production of other specific problems simultaneously studied, such as problematic video gaming [9] or social networking [10]. Both generalised and specific Internet use-related addiction problems seem to produce addictive symptomatology (i.e., the classic symptoms for substance use or gambling disorders) in a few users, together with functional physical or psychological impairments (i.e., when the online activity(-ies) negatively affects other areas of a user’s organism or lives; e.g., sight or academic/work facets), and distress (i.e., when the online activity(-ies) may reflect a maladaptive behaviour, failure in coping or adaptation processes).

**Figure 1:**
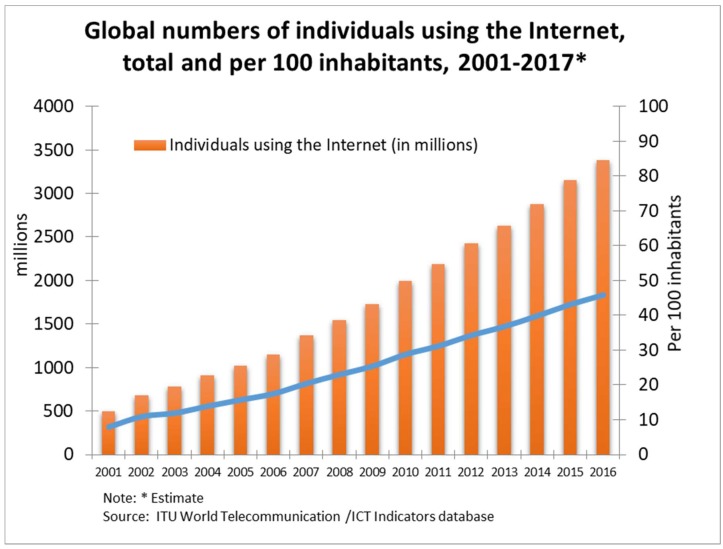
Evolution of global Information and Communication Technologies (ICT) from 2001 to 2017 (according to the International Telecommunication Union [ITU] World Telecommunications and its ICT indicators database).

Concerning statistics records, the International Telecommunication Union (ITU) has published its Information and Communication Technologies (ICT) Facts and Figures 2017 [11], showing the continuous worldwide expansion of Internet use through fixed or mobile subscriptions. For instance, in 2017, 3578 million individuals were using the Internet (compared to 495 million in 2001), 830 million being young people (i.e., 15–24-year-olds), which represents 80 per cent of the youth population in the 104 countries studied (see Figure 1).

Similarly, bibliometric evidence extracted from three ProQuest Central scientific databases (i.e., Health and Medical Collection, Psychology Database, and Public Health Database [12]) shows this impactful increase in research on Internet use-related addiction problems during the last two decades (1995–2017), which have especially risen from the beginning of the 21st century [13]. 

**Figure 2:**
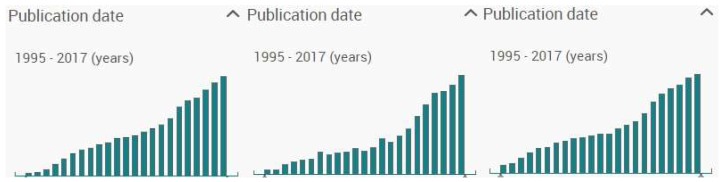
Evolution of Internet use-related addiction problems from 1995 to 2017 (according to the ProQuest Central databases on Medicine, Psychology, and Public Health). Note: The diagrams of bars are ordered from left to right as follow: Internet addiction, problematic gaming, and problematic social networking.

Indeed, an advanced search was conducted to observe the increase of production on these addiction problems. The procedure for the search of Internet addiction production was: It was introduced the terms ‘Internet addiction’ OR ‘problem* Internet use’ OR ‘pathological Internet use’ OR ‘excessive Internet use’ (to cover the main terminology used in Internet addiction). Simultaneously, the options to refine the search were: ‘Peer reviewed’ (i.e., to ensure the outputs were articles from official editorial processes), ‘Exclude duplicate documents’ (i.e., to avoid any article is detected more than once through the databases selected), ‘Show additional terms included in the search’ (to suggest and add alternative terminology used in relation to the terms selected), and a period from 1 January 1995 to 31 December 2017. A total of 116,455 outputs were obtained, showing a growing tendency in its bibliometric production, where each bar corresponded to the number of articles published in a year, going from 146 records in 1995 to 11,630 in 2017 (see Figure 2, first plot). The same procedure was used with the other two specific online behavioural addiction problems studied. Concerning problematic gaming (i.e., ‘gaming addiction’ OR ‘problem* gaming use’ OR ‘pathological gaming use’ OR ‘excessive gaming use’ OR ‘gaming disorder’ OR ‘internet gaming disorder’), 7246 outputs were addressing problems within video gaming (e.g., computer gaming, digital or electronic game addiction). These ranged from 35 records in 1995 to 839 in 2017, with a progressive increment from 2010 and especially from 2014 (after the inclusion of ‘Internet gaming disorder’ (IGD) in the appendix of the DSM-5 by the APA [6]; see Figure 2, second plot). Similarly, problematic social networking obtained the most outputs in the search: A total of 202,045 (using as keywords: ‘social network* addiction’ OR ‘problem* social network* use’ OR ‘pathological social network* use’ OR ‘excessive social network* use’ OR ‘social network* disorder’); from 1474 in 1995 to 18,595 in 2017; with a continuous growth accelerating since 2008 (i.e., the year that Facebook started to be internationally used with the design and functionalities most known; see Figure 2, third plot).

Research into these generalised and specific potential behavioural addictions has provided fundamental advances in clarifying conceptualisation and operationalisation within the spectrum of these problems. 

Firstly, Davis in 2001 [14] introduced one of the few existing theoretical models on Internet addiction. His cognitive–behavioural model of pathological Internet use (PIU) proposed dividing the problem into two types: Specific Internet use-related addiction problems (SPIU, referring to the condition in which an individual pathologically uses the Internet for a purpose; e.g., online gaming) and generalised PIU (GPIU, referring to the global set of online behaviours). The basic idea was that cognitive distortions (e.g., thoughts such as ‘the Internet is the only place where I am respected’) were automatically and unintentionally enacted whenever a stimulus associated with the Internet was available, resulting in emotional or behavioural outputs (i.e., GPIU or SPIU symptoms). Thus, maladaptive cognitions impact SPIU or GPIU, but the latter type was found to be more complicated, as several external factors could be the cause (e.g., social isolation, lack of social support), with negative consequences, such as procrastination and other daily functioning problems (e.g., putting off responsibilities to be online without a directive purpose). To the authors’ knowledge, no previous author has asked yet why the same external problems would not be present in generalised and specific Internet-use addition problems. This is a central question, as few works have addressed these factors in SPIUs, which are precisely the ones being treated, studied, and even recognised internationally in the case of gaming disorder.

Between 2002 and 2010, Caplan [15,16] tested and updated this model for GPIU, indicating some users had a preference for online social interactions and used the Internet for mood regulation, which predicted deficient online self-regulation (i.e., compulsive Internet use and a cognitive preoccupation regarding the Internet). Subsequently, Haagsma and colleagues in 2013 [17] tested the model for a SPIU (i.e., online gaming) into an adolescent population and obtained similar findings. However, although these studies showed a few readjustments in Davis’ model (e.g., internal factors, such as a preference for online interaction, online mood regulation, and online deficient self-regulation could predict both GPIU or SPIU), there is still a need for more theoretical development on generalised and specific online problems, especially addressing external (i.e., environmental) factors and structural (i.e., video games) factors. 

Secondly, Griffiths in 2005 [18] adapted Brown’s [19] proposal for gambling and developed the component model of addiction to operationalise the common features among substance and behavioural addictions. The addictive problem is formed in a biopsychosocial framework (i.e., as a consequence of individual, situational, and structural factors) and could be defined by six components (i.e., symptoms; see Table 1). Recently, Griffiths [20] has clarified common components are essential keys to delineate behavioural and substance addictions, as although different addictions have idiosyncrasies, these also have similarities (i.e., components), which are critical to the behaviour being labelled as an addiction. Thus, the six components need to be endorsed to operationally define an addiction (e.g., independently if generalised or specific problems). However, in public and mental health organisations (e.g., APA), and in their respective health manuals (e.g., the DSM), the components are usually articulated as criteria. Thus, to diagnose a disease or a mental health illness, the patient has to endorse a number of criteria (e.g., in the IGD proposed by the DSM-5, it is five out of nine). This quantitative approach has generated concern to estimate the prevalence of potential problematic users, as it has been dependent on several factors (e.g., the criteria, psychometric tools). Moreover, some criteria are more prominent and relevant than others, which have been tackled by few authors. 

Thirdly, concerning Internet addiction, Tao and colleagues [21] proposed renowned diagnostic criteria for Internet addiction (see Table 1). This had an operationalisation which introduced specifications: First, the relevance of symptoms (i.e., some being more important than others); second, the timing for the problem course (i.e., the addictive problem requires a period over which to be developed); third, exclusion tactics to diagnose the problem (i.e., the addictive problem should be differentiated from other disorders); fourth, the importance of the functional impairment (i.e., the addictive problem affects users’ real-life, impeding at least a facet of a user’s functioning). In concrete terms, they stated preoccupation and withdrawal should always be present, as these are the most important symptoms. Subsequently, at least one of the other symptoms should also be present (i.e., tolerance, persistent desire and difficulty to control it, continued use disregarding harmful consequences, loss of interests, or alleviation of negative emotions). The exclusion criteria were psychotic or bipolar I disorders, together with the need for a clinical impairment criterion (in other duties or relationships), and course the criterion of using the Internet excessively for at least three months for six hours per day as entertainment. Tao and colleagues’ proposal [21] together with substance use disorder (SUD) and gambling disorder criteria were taken as a source by the APA to propose the IGD criteria [22], which slightly differ in the symptoms, time of development (i.e., a year), and exclusion criteria (other excessive Internet uses). The IGD criteria are: (1) Preoccupation, (2) withdrawal, (3) tolerance, (4) reduction/stop, (5) giving up other activities, (6) continuing despite problems, (7) deceit/cover-up, (8) escaping adverse moods, (9) risk/loss of relationships/opportunities (the wording proposed for these criteria has been introduced in Table 1). Nevertheless, the field has recently been characterised by intense debate on whether and how IGD or Internet addiction (among other addiction problems) should become official disorders. These debates are central to the question of how general versus specific behavioural addiction forms. 

Thus, critiques of the component model, Internet addiction (i.e., as GPIU), or IGD (i.e., as SPIU) have emerged [23,24]. For instance, Griffiths’ and Tao’s proposals were criticised by Van Rooij and Prause [23], as they considered that the evidence base was not enough to support the diagnostic of the generalised Internet addiction as a behavioural addiction. They suggested studying common unpaired dimensions with the support of neuroimaging proofs, and identifying the changes in the rewarding element of using the Internet (e.g., if users are responding to the hedonic reward indistinctly if it is sex, drugs, or online behaviours, and how this response changes their usage pattern) [23]. Similarly, Kardefelt-Winther [24] criticised the IGD criteria for being too adhered to other previous addictive disorders included in the fourth DSM (DSM-IV), rather than capturing the phenomenology of online gaming; he argued some criteria were weak to diagnose SPIU (e.g., preoccupation, withdrawal, loss of interests, and tolerance; especially the later one, which belongs to SUDs). Moreover, it is worth noting how a relevant component, such as salience, which originally covered person’s thinking, feelings, and behaviours [18,19] has been reduced to a sole cognitive facet in this century’s research and clinical works; even the Davis model was only based on the cognitive approach of the maladaptive behaviour of Internet addiction.

Fourthly, Charlton and Danforth in 2007 [25] stated the distinction between Internet use-related addiction problems and high online engagements through specific components (i.e., criteria). They established the difference between core and peripheral criteria for behavioural addictions, initially performed for the GPIU, and subsequently applied to an SPIU: Online gaming (i.e., massively multiplayer online game playing [MMORPG]; e.g., Asheron’s call; see Table 1). The core criteria which defined an addictive problem were: Conflict (with other personal activities or other persons), withdrawal, relapse and reinstatement, and behavioural salience; while peripheral criteria were present in nonproblem online users (i.e., high-engagement users): Cognitive salience (e.g., preoccupation), tolerance, and euphoria. Moreover, time spent on online gaming was positively associated with those who highly scored on the core criteria. A study recently carried out by Lehenbauer and Fohringer [26] has found similar results regarding online gaming. They adapted the version of a previous MMORPG (i.e., World of Warcraft), and found differences between highly-engaged and addicted gamers to the same core versus peripheral criteria, together with more time spent on the core criteria (i.e., addicted gamers were gaming 30 h per week, while highly-engaged players played around 20 h). Furthermore, this study also showed the quality of life for addict gamers was significantly lower than highly-engaged players, especially in physical and psychological health indicators. 

For problem Internet and gaming addictions, other research has been undertaken this decade and provided evidence about other potential behavioural addictions which could be classed into this spectrum of online addiction problems [27]. However, the existing theoretical studies (e.g., critiques, reviews) have scarce theoretical development, and only a few of the classic studies have developed attempts of theoretical models (e.g., Davis’ model [14]). Studies are usually empirical and use a quantitative approach (e.g., surveys), while qualitative or mixed methods approaches are still scarce, as in other complementary fields, such as behavioural or educational sciences [28,29]. At present, there is a need for knowledge on the phenomenology (i.e., nature) of these problems and more theoretical development. Young developed the latest published study addressing the phenomenon of Internet addiction in 2004 [30], eight years after she coined the term [2,5]. She provided awareness about the nature of Internet use and its potential abuse, as a decade ago this phenomenon had not been identified and defined yet [31,32]. Similarly, recent research has been published, tackling the phenomenology of traditional gaming versus gaming addiction through insights from the gamers’ perspective [33], especially in younger generations. Thus, GPIU and SPIU (gaming) might become emerging public health problems as, according to Grant, Schreiber, and Odlaug [34], behavioural addictions are characterised by the inability to resist a drive, resulting in actions that are harmful to oneself and others. 

There is a need to cover the gap to start ascertaining the addictive nature of these Internet use-related addiction problems; especially when a public health organisation, such as the WHO, has included ‘gaming disorder’ as a behavioural addiction into the category for ‘Disorders due to substance use or addictive behaviours’, subcategory ‘Disorders due to addictive behaviours’, together with gambling (WHO, 2018 [7]). Furthermore, it seems that while other potential addictive problems connected to technological uses are still under investigation by academic and clinical professionals, there is limited evidence to consider their official recognition yet. This is the case of social media or social networking addiction, where a small number of controversial studies have reported these problems related to the maladaptive use of these media as entertainment and communication tools [35,36]. However, although a few psychometric instruments have been developed to measure this new phenomenon (based on previous addictive criteria, such as SUDs or IGD [37,38]), the evidence is limited and concentrated on adolescents and young adults. 

On the other hand, it seems to be established that the existing scales which have been developed in the field of technological (behavioural) addictions have usually been developed through other current addiction criteria and validated using student community samples. An outstanding scale is the compulsive Internet use scale (CIUS [39]), which has been recognised as one of the most psychometrically stable tools (e.g., similar factorial structure among several language adaptations) to measure both generalised and specific (i.e., cybersex) use-related addiction problems. In this study, this scale has been selected to quantitatively measure the phenomena of GPIU (i.e., Internet) and SPIUs (i.e., gaming and social networking), respectively (see Table 1). Indeed, psychometrically, the CIUS has excellent reliability, a unique factor structure demonstrated by exploratory and confirmatory approaches, and has shown measurement invariance [39]. However, as the purpose of this paper is to go further in depth and observe the commonalities and differences among GPIU versus SPIUs, a qualitative measure was performed by interviewing participants potentially classed as problem Internet, gaming, and/or social network users. This strategy required a mixed methods approach of a community sample, accomplishing the following methodological requirements provided below. 

Thus, the principal aim is to understand adults’ online uses in their personal sphere (i.e., non-academic or professional) to ascertain if these behaviours could be classed as GPIU and/or SPIU and to know about the problems from those who could be categorised as potential problem users. This is articulated in a four-fold specific aim: (i) To validate the CIUS adapted to these three Internet use-related addiction problems (i.e., Internet, gaming, and social networking) to compare them; (ii) to estimate the prevalence of potential problem Internet, gaming, and social networkers users to explore them by sociodemographic variables and addictive symptomatology; (iii) to examine potential problem users’ knowledge, experiences, and perceptions with regard to the nature and development of these problems; and (iv) to know their opinion on IGD criteria adapted to the GPIU and SPIUs studied. 

**Table 1:** Comparison of components, criteria, criterions in GPIUs and SPIUs proposals.

Components/Criteria	Subcomponents/Criterions	GPIU (Addiction; Griffiths, 2005 [18])	GPIU (Internet addiction; Tao et al., 2010 [21])	SPIU (IGD; APA, 2013 [22])	SPIU (online gaming; Charlton & Danforth, 2007 [25])	GPIU (CIUS; Meerkerk, Van Den Eijnden, Vermulst, & Garretsen, 2009 [39])
Salience [18,39], Preoccupation [21,22,25]	Cognitive salience [25,39]	When the activity becomes the most important thing and dominates person’s thinking, feelings, and behaviours	A strong thinking ongoing online	Do you spend a lot of time thinking about games even when you are not playing, or planning when you can play next?	I rarely think about playing when I am not using a computer	6. Do you think about the Internet, even when not online? 7. Do you look forward to your next Internet session?
	Behavioural salience [25,39]				I often fail to get enough sleep/miss meals because of playing	4. Do you prefer to use the Internet instead of spending time with others (e.g., partner, children, parents)?
Mood modification [18,25], alleviation of negative emotions [21,39], deceit or cover up [22] or escaping adverse moods [22,39]	Manage tension	Subjective experience as a consequence of engaging in the activity to increase or decrease tension to escape, or disconnect	Being online to escape or being relieved	Do you lie to family, friends or others about how much you game, or try to keep your family or friends from knowing how much you game?	I often experience a buzz of excitement while playing	12. Do you go on the Internet when you are feeling down?
	To escape or relieve			Do you game to escape from or forget about personal problems, or to relieve uncomfortable feelings, such as guilt, anxiety, helplessness or depression?		13. Do you use the Internet to escape from your sorrows or get relief from negative feelings?
Tolerance [18,21,22,25]		The need to increase amounts of the activity to achieve the preceding pleasant effects	Marked increase in online use to achieve satisfaction	Do you feel the need to play for increasing amounts of time, play more exciting games, or use more powerful equipment to get the same amount of excitement you used to get?	I tend to want to spend increasing amounts of time playing	
Withdrawal [18,21,25,39]		Unpleasant feeling states or physical effects when the activity is reduced or stopped	Dysphoric mood, anxiety, or boredom after days without online activity	Do you feel restless, irritable, moody, angry, anxious or sad when attempting to cut down or stop gaming, or when you are unable to play?	When I am not playing, I often feel agitated	14. Do you feel restless, frustrated, or irritated when you cannot use the Internet?
Conflict [18,25,39], loss of interests [21], give up other activities [22]	Intrapersonal [18,25,39], clinical impairment [21]	Conflicts from within the individual themselves		Do you lose interest in or reduce participation in other recreational activities (hobbies, meetings with friends) due to gaming? Do you risk or lose significant relationships, or job, educational or career opportunities because of gaming?	My social life/work has sometimes suffered because of my playing	8. Do you think you should use the Internet less often? 10. Do you rush through your (home) work in order to go on the Internet?
	Interpersonal [18,39], loss of interests and clinical impairment [21]	Conflicts between the addict and those around them	Online use substitutes (e.g., hobbies)		Arguments have sometimes arisen at home because of the time I spend playing	3. Do others (e.g., partner, children, parents) say you should use the Internet less? 11. Do you neglect your daily obligations (work, school, or family life) because you prefer to go on the Internet?
Relapse [18] and relapse reinstatement [25]		Tendency for repeated reversions to earlier patterns of the activity to be quickly restored after time of abstinence or control			I have made unsuccessful attempts to reduce the time I spend playing	
Persistent desire and difficulty to control it [21,39], reduction/stop [22,39]			Not being able to maintain a regular online usage pattern	Do you feel that you should play less, but are unable to cut back on the amount of time you spend playing games?		1. Do you find it difficult to stop using the Internet when you are online? 2. Do you continue to use the Internet despite your intention to stop? 5. Are you short of sleep because of the Internet? 9. Have you unsuccessfully tried to spend less time on the Internet?
Continued use disregarding harmful consequences [21,22]			Being online even causing psychological or physical harm to oneself	Do you continue to play games even though you are aware of negative consequences, such as not getting enough sleep, being late to school/work, spending too much money, having arguments with others, or neglecting important duties?		

Note: GPIU = Generalised Problematic Internet Use; SPIU = Specific Problematic Internet Use; IGD = Internet Gaming Disorder; APA = American Psychiatric Association; CIUS = Compulsive Internet Use Scale.

## 2. Materials and Methods

Permission to conduct this study was obtained from the ethics committee of the Psychological Science Research Institute (IPSY) at the Catholic University of Louvain (UcL; Belgium) in 2014. Respondents participated in leading research, which builds upon the Tech Use Disorders (TUD; [40]) project, carried out between 2014 and 2016. The qualitative part was undertaken at UcL, which is the main reason only Belgian results have been included in this paper. None of the data analysed in this paper have been used in any other article from this Marie Curie Intra-European Fellowship (FP7-PEOPLE-2013-IEF; ID 627999 [40,41,42,43,44]).

### 2.1. Design

Following the notation proposed by Morse [45] for the mixed methods designs, and according to the combination of data collection strategy (i.e., sequential implementation/time orientation) and priority (i.e., equal weight/emphasis) [46], a partially mixed sequential equal status (‘QUAN→QUAL’) design was used. The purpose of this design was to integrate in the discussion of the present study two different types of data [47,48,49] to clarify and illustrate the results obtained with the quantitative method by applying the qualitative one (i.e., whereby the interviews may help to evaluate and interpret the psychometric results obtained, estimating potential problem users who excessively perform a general or a specific online use(s)). A few of the previous studies on problematic online gaming have recently requested and undertaken this strategy [33] or the qualitative approach [50], but more should be done to cover the methodological gaps of finding what is behind the phenomena usually extracted from a sole psychometric measure. Another complementary purpose was to enable expansion (i.e., seeking to analyse and explore different facets of the GPIU and SPIU phenomena studied; e.g., to know if they seem independent or are interconnected, and in what sense). The mixed methods research in this study attempts to look for the way both methods complement each other to obtain a more productive, realistic, and detailed understanding of Internet addiction (as GPIU), online gaming disorder (as SPIU_1_), and maladaptive use of social networks (as SPIU_2_). 

### 2.2. Participants and Procedure

#### 2.2.1. Quantitative 

The study online surveyed a convenience sample from Belgian higher education environments with 581 adult participants (85% originally francophone; 25.5% male; age range 18–79 years, mean (*M*) age 26.9 years, standard deviation (*SD*) = 12 years), with student and staff members who voluntarily agreed to participate. Participants had an information sheet and consent form on the first page of the survey and they provided informed consent and voluntarily participated following assurance of confidentiality and anonymity. The invitation to join the online survey used three recruitment strategies: Undergraduates’ lectures, master or doctoral supervisions; via electronic requests or pools in online academic environments; and web sites or quick response code advertisements. 

#### 2.2.2. Qualitative 

Participants who scored equal or over 21 in each CIUS [51] were invited to interviews, as they could potentially be classed as problem users. They were usually university students (except one who was an employee), all speaking fluent French. The invitation to participate in the qualitative part of the study was included at the end of the survey. The author contacted all participants who achieved the inclusion criteria reported and eight (1.4% of the overall sample) agreed to engage in the interview with an economic compensation (20 euros). They received an information sheet, provided informed consent by signing an agreement, and voluntarily participated following assurance of confidentiality and anonymity using pseudonyms. Interviews had a duration between 45 min and an hour, which took place at the IPSY (UcL). Permission was received for audio-recording. 

### 2.3. Instrument and Analytical Strategy

#### 2.3.1. Quantitative

The quantitative method was an online survey developed using Qualtrics which comprised: (i) Sociodemographics (gender, age, civil status, occupation status); (ii) online usage time (i.e., usual minutes per week day, and usual minutes per weekend day); (iii) technologies (i.e., fixed/desktop computer, laptop/notebook, tablet, smartphone, fixed game console or nonportable console, portable game console, television, other devices); (iv) main online activity used (i.e., emails (e.g., Google Mail, Oulook.com), messaging and chat (e.g., Skype, Hangouts), maintaining a blog (e.g., WordPress, Tumblr), online videos or streaming (e.g., YouTube, Netflix), downloads (e.g., music, movies), reading (e.g., newspapers, e-books), search for specific information (e.g., weather forecasts, city maps), casual games (e.g., Candy Crush, Farmville), solo video games (e.g., Dragon age, Assassin’s Creed), vehicle simulation games (e.g., Euro Truck Simulator, Flight Simulator), strategy and management games (e.g., Age of Empire, StarCraft, Civilization), sport games (e.g., Pro Evolution Soccer, Virtua Tennis), shooting games online or first-person shooter (FPS; e.g., Call of duty, Planet side 2), multiplayer online battle arena (MOBA; e.g., League of Legends, Dota 2), MMORPG (e.g., World of Warcraft, Guild Wars 2), online gambling (e.g., PlayHugeLottos.com, OnlineBingo.eu), sport bets (e.g., PMU, horseracing, football), poker online (e.g., PokerStars.com), online casino (e.g., Casino Online, Blackjack), online slot machines, dating sites (e.g., Match, Meetic), erotic sites (e.g., Erotica), pornographic sites (e.g., Youporn), online shopping (e.g., eBay, Amazon), social networks (e.g., Facebook, Twitter)); and (v) the psychometric scales assessing generalised and specific Internet use-related addiction problems reported. Other studies have used and analysed other sections of this survey (e.g., other questionnaires in other samples from different countries or group ages), usually related to problematic mobile phone use and gaming [41,42,43,44]. 

Sociodemographics examined gender, age, relationship status (i.e., single, in a relationship, legally cohabitating, married, separated, divorced, or other), profession (i.e., student, employed, unemployed, retired, housewife/husband, self-employed, or other). The CIUS [39] measures general problem or compulsive Internet use (i.e., Internet addiction). It is based on the fourth DSM criterion for SUDs and pathological gambling. It contains 14 items rated from 0 “never” to 4 “very often”. Scores ranged from 0–56, with the higher scores meaning to the more top potential generalised compulsive online use. The original scale in Dutch showed factorial, content, and concurrent validity and excellent reliability (Cronbach α between 0.89 and 0.90), as did the French adaptation used [52], which obtained similar outstanding psychometric properties (i.e., Cronbach α = 91). In this study, the original item had three options to be answered: General Internet use, online gaming, and social networking. For instance, item 1 (in French: ‘A quelle fréquence, trouvez-vous difficile d’arrêter d’utiliser Internet pendant que vous êtes en ligne (c’est à dire s’arrêter, stopper l’activité)*’*; in English: How often do you find it difficult to stop using the Internet when you are online?) had three responses: (a) For Internet in general (CIUS), (b) for online gaming (CIUS-G), and (c) for social networking (CIUS-SNS). Thus, participants completed each item with these online uses. This innovation allows to assume more than one online use could be present simultaneously for any participant.

All statistical analyses were performed using IBM SPSS (version 23) software (IBM Corp. Released, Armonk, NY, USA) and a significance level of *p* < 0.05. Concerning the psychometric properties, the factor validity of each scale adaptation was assessed by exploratory factor analysis (EFA) using the principal components technique, with the Kaiser–Mayer–Olkin index (KMO) and Bartlett’s test of sphericity to confirm the adequacy of the sample and procedure, respectively. The rationale for using the EFA is no study has adapted the CIUS to measure other specific Internet use-related addiction problems until the present, as far as the author is aware. The analysis yielded one factor, which is consistent with the theory and previous research using the CIUS [39]; with eigenvalues above 1 (factor loading > 0.4) to obtain an acceptable factor based on its explained variance. Internal consistency was estimated through the Cronbach’s alpha coefficient, and an item analysis was performed to compare all CIUS forms. Construct and content validity were obtained through associations of the total score with several indicators (i.e., time spent online using the Internet in general, or spent mainly gaming or mainly on social networks; or activities usually used by each of the CIUS studied). Comparisons between CIUS total scores and sociodemographic variables (i.e., gender and age groups) were performed through *t* and *U* Mann–Whitney tests. The proposed cut-off score reported by Guertler and colleagues [51] was chosen (i.e., score of 21 out of 56) to estimate the prevalence of potential problem users. However, caution should be considered, as present cut-offs still do not represent a threshold for clinical relevance and impairment. The sum of all potential problem users was computed to know how many participants could have one (or more) of the problems studied, together with the items and symptoms endorsed. Finally, to compare core versus peripheral symptoms [25,26] in those classed as potential problem users, the three CIUS were divided in two subgroups, according to Meerkerk and colleagues’ [39] and Charlton and Danforth’s [25] proposals (core symptoms: Items 3, 4, 5, 8, 10, 11, 14; peripheral symptoms: Items 1, 2, 6, 7, 9, 12, 13). Only an item (5) was modified from Meerkerk’s proposal (i.e., from ‘loss of control’ was moved to ‘intrapersonal conflict’). 

#### 2.3.2. Qualitative 

The interviews were undertaken and transcribed. The methods selected for the qualitative data analysis were two [53]: First, thematic analysis [54,55] with an etic coding strategy (i.e., coding from the perspective of the observer based on the literature) was used for examining perceptions and attitudes about potential Internet use-related addiction problems (causes, development, consequences, factors). Second, content analysis [56,57] to focus at a more micro level to provide both counts and opinions on IGD criteria proposed by the APA applied to the three problems studied. Both methods belong to qualitative descriptive phenomenological approaches [53], with a lower level of interpretation, but higher detail on the description of the meanings (i.e., focused on the knowledge that can originate and establish new definitions and substantial findings). At this stage of the development of the field, detail is needed on the nature of these problems. Synthetically, thematic analysis constitutes a purely qualitative approach used for identifying, analysing (codes), reporting patterns (themes) within textual data (i.e., involving the search for and identification of common threads), and interpreting aspects of the research question [56]. On the other hand, content analysis is a systematic coding and categorising approach used to explore text in detail to find patterns of words used, their frequencies (counts), their relationships, and their structures, which will allow answering the research questions [58]. 

## 3. Results

### 3.1. Quantitative 

#### 3.1.1. Participants Characteristics: Sociodemographic, Technologies Usage Patterns, and Online Activities

The majority of the sample (71.8%) were students (only 17.3% were employees, and the rest were 2.9% self-employed, 2% retired, 1.1% unemployed, 0.05% housewives or househusbands, among other). Half of the sample (58.2%) were single (36.5% were couples/in legal cohabitation/married, 4.9% separated/divorced, among other), without children (only 15.3% had progeny); their maximum education level was above all between secondary (59.8%) and higher education (38.8%). The participants (99.8%) had an Internet connection where they lived, had at least a computer (desktop or laptop; 97.5%) and a mobile phone (99.3%: without Internet access (25.1%) or a smartphone (74.2%)), and a third had (34.7%) a Tablet (e.g., iPad, Samsung galaxy tab). In all technologies, they usually had monthly contracts (i.e., Internet at home 95.5% of the sample, in the mobile phone 75.1%, and for the Tablet 35.3%). 

#### 3.1.2. Psychometric Properties of the French Generalised and Specific CIUS in Belgium

The CIUS as a GPIU psychometric tool was previously validated [39,52], but not the CIUS as SPIUs scales in gaming and social networking. In this study, the three measures showed factorial validity (i.e., CIUS: KMO = 0.91, χ^2^_(91)_ = 2673.09, *p* < 0.001; CIUS–G: KMO = 0.93, χ^2^_(91)_ = 5223.13, *p* < 0.001; CIUS–SNS: KMO = 0.93, χ^2^_(91)_ = 3519.31, *p* < 0.001) and excellent internal consistency (see Table 2; where the descriptive, Cronbach alpha coefficients, and correlations between the three CIUS are reported). It is worth noting the high positive association between CIUS and CIUS–SNS, and the low positive association between CIUS and CIUS–G; however, between the two specific uses (CIUS–G and CIUS–SNS), there is no correlation. 

**Figure 3:**
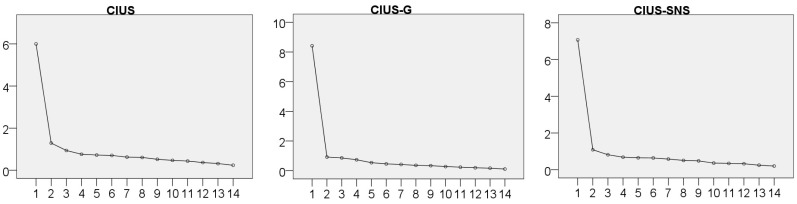
Scree plots from the original CIUS (GPIU), the adaptation for gaming (CIUS–G; SPIU_1_), and the adaptation for social networking (CIUS–SNS; SPIU_2_); in the y axis the eigenvalues, and in the x axis the component numbers.

The CIUS yielded one factor (i.e., generalised problematic Internet use), which explained 48.86% of the total variance with loads to this sole factor between 0.85 (item 14) to 0.72 (item 10); such as the CIUS–G (i.e., specific problematic (video) gaming) that explained 60.12% with loads to between 0.69 (item 9) to 0.84 (item 7), and the CIUS–SNS (i.e., specific problematic social networking) that explained 50.50% with loads to between 0.58 (item 4) to 0.79 (item 2). The scree plots of the three versions show the one-factor of these adaptations (see Figure 3), which allows to compare items by descriptive, correlations, and factors loadings (see Table 3). 

Regarding descriptive results per item in each version, CIUS and CIUS–SNS have higher scores than CIUS–G. The higher punctuations in all of them being for items 1, 2, and 12 (which were associated with loss of control and mood modification symptoms [39]). However, differences between adaptations emerged in the lowest scores: Item 9 on Internet and gaming (loss of control [39]), and item 4 in social networking (preoccupation [39]). Concerning the correlation of each item with the total correlation of each adaptation, all CIUS presented high positive correlations between each item and the total score, especially the gaming version (*r* between 0.63 and 0.80). Concerning factor loading, all were greater than 0.5; by order from higher to lower scores: Gaming (minimum 0.69), social networking (minimum 0.58), and Internet (minimum 0.53). Consequently, when dividing the CIUS scores by core and peripheral symptoms, descriptive results were higher for peripheral ones (i.e., Internet use in general (*M* = 10.34, *SD* = 5.87), social networking (*M* = 9.32, *SD* = 6.77), and gaming (*M* = 3.06, *SD* = 5.25)) than the core symptoms (i.e., Internet (*M* = 7.38, *SD* = 5.32), social networking (*M* = 6.23, *SD* = 5.70), and gaming (*M* = 2.04, *SD* = 4.12)). 

Furthermore, associations between the time indicators and the three CIUS total scores strengthen the construct validity (see Table 4). In this regard, almost all the time variables were significantly associated with the three CIUS, but differently according to the technology. For instance, moderate correlations on the Internet and gaming addiction problems were present in almost all technologies, while social networking addiction problems were present, especially using smartphones. Interestingly, a device pattern emerged as significant associations were quite heterogeneous; for instance, all technologies were associated with time per days (weekday or weekend day) when using Internet in general, but when gaming, only computers and tablets were correlated with time per days, and when social networking, only computers and smartphones were associated with time spent on a day. However, the maximum time spent as leisure was usually on computers.

Similarly, content validity was also established through associations to CIUS total scores and across all the online activities as follows: Firstly, the CIUS (GPIU) with ‘messages and chats’ (*r* = 0.19, *p* < 0.001), ‘online videos and streaming’ (*r* = 0.17, *p* < 0.001), ‘Twitter’ (*r* = 0.16, *p* < 0.001), ‘Facebook’ (*r* = 0.14, *p* < 0.001), ‘Instagram’ (*r* = 0.12, *p* < 0.05), ‘Hi5’ (*r* = 0.11, *p* < 0.05), ‘blogs’ (*r* = 0.10, *p* < 0.05), and ‘porn sites’ (*r* = 0.09, *p* < 0.05). Secondly, the CIUS–G (SPIU_1_) was associated to the following games: ‘MOBA’ (*r* = 0.45, *p* < 0.001), ‘MMORPG’ (*r* = 0.43, *p* < 0.001), ‘Solo games’ (*r* = 0.42, *p* < 0.001), ‘FPS’ (*r* = 0.39, *p* < 0.001), ‘games strategy’ (*r* = 0.33, *p* < 0.001), ‘casual games’ (*r* = 0.23, *p* < 0.001), and ‘simulation games’ (*r* = 0.22, *p* < 0.001), together with porn sites (*r* = 0.24, *p* < 0.001), and reading (*r* = 0.1, *p* < 0.05). Lastly, the CIUS–SNS (SPIU_2_) was related to the following social networks: ‘Facebook’ (*r* = 0.36, *p* < 0.001), ‘Instagram’ (*r* = 0.18, *p* < 0.05), and ‘Twitter’ (*r* = 0.14, *p* < 0.001), including messages and chats (*r* = 0.23, *p* < 0.01), downloading (*r* = 0.2, *p* < 0.001), streaming (*r* = 0.18, *p* < 0.001), and blogging (*r* = 0.1, *p* < 0.05). 

#### 3.1.3. Prevalence of the French Generalised and Specific CIUS in Belgium

In this study, no statistical difference was found between the original CIUS total score per gender (*t*_(469)_ = −0.08, *p* = 0.94; male: *M* = 17.65, *SD* = 11.05; female: *M* = 17.75, *SD* = 10.48), but differences between gender were found in the specific CIUS: CIUS–G (*U* = 13,420, *p* < 0.001; male: *M* = 10.05, *SD* = 12.30; female: *M* = 3.68, *SD* = 7.42), CIUS–SNS (*U* = 13,945, *p* < 0.001; male: *M* = 11.25, *SD* = 10.55; female: *M* = 16.78, *SD* = 12.03). With respect to the age groups, young adults (i.e., 18–39 years old) compared to middle-aged adults (i.e., 40–65 years old) presented significant differences in all measures: CIUS (*t***_(_**_459**)**_ = −5.54, *p* < 0.001; young: *M* = 18.58, *SD* = 10.35; middle-aged: *M* = 10.41, *SD* = 8.85), CIUS–G (*U* = *9160, p* < *0.05*; young: *M* = 5.34, *SD* = 9.37; middle-aged: *M* = 2.81, *SD* = 6.42), and CIUS–SNS (*U* = 4493.5, *p* < 0.001; young: *M* = 17.05, *SD* = 11.70; middle-aged: *M* = 5.89, *SD* = 7.66). 

Concerning GPIU, taking into account the overall 471 participants as potential problem users, there were 176 potential Internet addicts (37.4%: 8.1% males and 29.3% females, χ^2^_(1)_ = 23.58, *p* < 0.001; 39.8% young and 13% middle-aged, χ^2^_(1)_ = 14.79, *p* < 0.001), 51 potential problematic online gamers (10.8%: 5.3% males and 5.5% females, χ^2^_(1)_ = 0.08, *p* = 0.78; 11.3% young and 3.7% middle-aged, χ^2^_(1)_ = 2.95, *p* = 0.09), and 153 problematic social networkers (32.5%: 3.4% males and 29.1% females, χ^2^_(1)_ = 18.09, *p* < 0.001; 32.4% young and 7.4% middle-aged, χ^2^_(1)_ = 23.58, *p* < 0.001). Finally, when computing the potential problem users, 49.7% of the sample has not classed in any user problem category, 23.6% had only one problematic online use, 23.1% had two potential online problems, and 3.6% had all the problems. 

From these three groups of problem users, descriptive and endorsements to each item (i.e., on the basis of answering ‘often’ or ‘very often’ should be considered) were computed (see Table 5). Interestingly, the highest scores and endorsement in all GPIU and SPIUs were in items 1 and 2 for the loss of control symptom, and item 12 for the mood modification symptom; while the lower scores and endorsements were more heterogeneous. For instance, item 3 (conflict) being lower in the problematic Internet (CIUS), but the less endorsed were items 4 (preoccupation) and 9 (loss of control); while in gaming (CIUS–G) item 9 (loss of control) was the one with lower score and the less endorsed by none potential problem gamer; lastly, item 4 (preoccupation) obtained the lower score and endorsement in problematic social networking. 

Finally, when comparing core and peripheral symptoms in each CIUS peripheral ones used to have higher punctuation than core ones in the potential problem users in GPIU and SPIUs (see Table 5). Furthermore, for those categorised as problem Internet users, moderate associations between time variables and core or peripheral items were significant in the time spent using Internet on a weekday (core: *r* = 0.19, *p* < 0.05; peripheral: *r* = 0.21, *p* < 0.05 ) or a weekend day (core: *r* = 0.23, *p* < 0.01; peripheral: *r* = 0.22, *p* < 0.05); while in problem social networkers there existed only an association between the core symptoms and weekend days (*r* = 0.25, *p* < 0.01), and none association existed in problem gamers (e.g., core: Time spent gaming in a weekday (*r* = 0.21, *p* = 0.19)). 

### 3.2. Qualitative 

#### 3.2.1. Participants Characteristics: Sociodemographic, Technologies Usage Patterns, and Online Activities

A total of 165 (out of 581) provided their contact details to participate in the qualitative part of this study; eight of them (4.85% of those who left this information) accepted the invitation to participate in an individual interview. Participants’ characteristics are reported in Table 6. 

Almost all participants were female Belgian young adults who scored as potential problem users of Internet in general (*n* = 5), gaming (*n* = 4), and/or social networking (*n* = 4). Therefore, half of them had mixed problem profiles, usually as excessive Internet in general and social network users, except one presenting all problems studied (i.e., GPIU, SPIU_1_, and SPIU_2_), who was a young male.

#### 3.2.2. Themes Related to Generalised Internet Problem, and Specific Gaming and Social Networking Problems 

##### Themes Related to Its Evolution: Causes, Development, and Consequences

Individuals’ experiences and reflections mapped into the central themes which are presented in Table 7, Table 8, Table 9 and Table 10 below by types of GPIU vs. SPIU.

The causes of excessively using specific online activities were diverse (see Table 7), and they could be summarised in three types of aetiological facets: the individual, the social, and the contextual ones. First, the individual aspect relates to avoiding boredom and to escaping reality (as a daily routine or because of a trauma). In the case of gamers, it seems there is a profile: they tend to be tech-savvy (i.e., study or work in informatics or the technologies sector) or really enjoy technologies (to be daily connected, multitasking, and managing studies/work and leisure), together with being a homebody person with an introvert personality, and to have a specific desire or mood to only play video games. By contrast, social networkers would like to have consistent updates in all areas and use SNS obsessively to scrutinise others’ lives and compare them with their own life. Second, the social facet seems to be related to sharing a hobby with others who have similar interests in technologies and online activities (e.g., relatives, boyfriend/girlfriend, friends from real life, virtual colleagues); gamers usually are highly committed to their guild and tend to supply (or complement) their social life through virtual social friendships developed (or maintained) through the game. It seems online users tend to build (or strengthen) bonds with those who share the same online activities and they are constantly connected through games and networks sharing information (e.g., about competitions, gossips). Third, the contextual facet is usually linked to having plenty of free time (e.g., because they are studying at secondary school or university, unemployed, in convalescence, or they are housewives/househusbands); a perceived ‘poor’ or ‘hostile’ external context (e.g., difficult relationships (or not fulfilling ones) with family, partner, colleagues), an environment where everybody is connected to the Internet (e.g., gamer guilds, Facebook profiles), being in a post-traumatic recovery (e.g., having lost a loved one(s), having lost a job), or feeling the need to consume technology when something new and appealing appears in the market (e.g., new video game, new social network, latest version). Interestingly, no quote was extracted from GPIU, and almost all were obtained from gaming.

The development of these Internet problems is described as a process, which starts during adolescence with an acquired enjoyable habit that slowly and unconsciously takes progressively more and more from the user (i.e., not only more time; by coming above all other life aspects of the user, such as feelings, emotions, cognitions, behaviours, relationships, activities). This gradual mining of the problematic online uses could be observed through the addictive symptomatology described by excessive users (see Table 8). 

Regarding salience, it is usually described covering emotional and cognitive facets (i.e., craving or preoccupation respectively; e.g., as a personal drive or desire to be connected or worrying, even being obsessive about personal or social online duties, such as updating your profile, tending to notifications in the SNS, or accomplishing guild expectations in a role-play game). Sometimes, it is associated with an unconscious need to be connected even when not possible, or an alleviation of negative emotions, such as wanting to escape or being ‘anesthetised’. It seems to be quite a usual symptom in the three problems studied. 

With regard to mood modification, salience is present in the GPIU and SPIUs studied, but it seems more prevalent in gaming. Gamers report using various kinds of games to produce different types of moods, such as MMORPG or FPS to induce tension, and casual games to relax; they state they balance their spirit in each moment through the games they choose to play. Similarly, social networkers, for instance, use Facebook to channel emotions (e.g., to share good news vs. to look for someone to cheer up). Networkers state they use SNS to share positive things (e.g., positive messages, images, videos). SNS contents seem to be positively biased, but networkers are aware and happy with it. However, sometimes, images or other information observed through the SNS could affect a user’s humour negatively (e.g., as the user could compare his or her life with others; especially in adolescence), and feel upset caused by negative feelings (e.g., jealousy, sadness). This could also happen with the online series that people watch in streaming too, usually used to relax (e.g., users could compare their lives, and this could affect them, especially when being an adolescent).

Tolerance is a symptom which only emerged in both SPIUs studied. In gaming, it seems to be linked to all types of continuous increments: Time, expertise, the sensation of progress, achievement, and advancement in the game to produce similar satisfaction or rewards. Therefore, the ‘dose’ is not only a quantity of time playing, it is also an increment of intensity and development of the gamer’s skills in the game, as well as the level of personal achievement in the game (e.g., score, level) or the level of social recognition by other gamers (e.g., status in the guild). Similarly, in social networking, the tolerance symptom could be understood for the continuous need and increment of publishing information (e.g., posts, pictures) about one’s own (or another’s) life, to observe social reactions (e.g., how many likes and/or comments the post received). This reinforces specific online behaviours, as the user self-perceives his or her life as a gamer or networker good enough to be valued by those who share the same online activity (e.g., through score comparisons, others’ life achievements). 

The other classic addictive symptom studied was withdrawal, which has usually been associated with negative feelings (i.e., frustration, irritability) when not being connected for gaming. This sense is directly linked to the need of connection after interruption or a forced stop. It seems being disconnected is being in severe discomfort, above all when the user is younger (e.g., an adolescent), alone at home (i.e., with plenty of time to play games), and a gamer (i.e., playing ‘big video games’, which usually are MMORPG, FPS, or MOBA). Thus, in the GPIU and SPIU related to SNS, withdrawal has not emerged as a subtheme.

Concerning conflicts, intrapersonal and interpersonal facets have emerged for all Internet use-related addiction problems. Gamers are the users who appeal more often to intrapersonal conflicts, as they play everywhere when a computer is available to them (e.g., university, work, or home), and when they supposedly should do other activities (e.g., attending a session in university, working in a workplace, or sleeping during night). However, social networkers have reported similar problems with depression, anxiety, and stress, as well as sleep or meal deprivation, which could affect their duties or relationships. Subsequently, there are problems with family, friends, or partners because of excessive online behaviours, which cause disputes, possible loss of contacts (e.g., boyfriend, girlfriend) or less of other relevant activities (e.g., studies, job). 

Concerning relapse, this only appeared once in a gamer interview, who expressed how rigid rules created by the guild could constrain the gamer and obligate him or her to play. If the player leaves the game, social pressure appears by the guild, as well as the loss of the feelings of being in a ‘virtual world’ created by the gamers during an extended period. Thus, the gamer continually returns to the game. 

The persistent desire and difficulty to control, reduce, or stop online behaviour is a common difficulty for all excessive Internet users. For instance, gaming is an extended hobby with an industry which supports periodic updates to play better and differently (e.g., characters, collections, the system of dropping). Moreover, the fact that some games reproduce ‘persistent worlds’ facilitates continuous playing (e.g., to compete). Similarly, the continued use with disregard of harmful consequences (e.g., physical problems or unhealthy attitudes or behaviours) is usually due to the habit already created to manage the user’s mood, user’s time, social relationships, etc. It seems essential to be continuously connected to the Internet, games, or SNS to maintain users’ personal and social life actively. 

Lastly, deceit or cover-up has only appeared in problematic gaming, as gamers usually report they live a ‘secret double life’, the one in the ‘real world’ and the one in the ‘virtual world’ (i.e., the game(s)). Gamers are quite aware they invert time and effort in the games; contrary to other established addictive behaviours, gamers work hard and actively to maintain their gaming behaviour. One reason is other gamers (e.g., the guild) when playing in the ‘big games’ share their own online spaces and codes to constantly communicate (even if they know themselves or not in ‘real life’, as sometimes gamers are from other countries or cultures). Thus, deception is not only hiding what is consumed through technology to others (e.g., hiding playing games at night to their parents), it is a more complex issue (i.e., managing languages, identities, relationships, and duties in different ‘worlds’). In this sense, they express that those who do not play games usually cannot understand them; they directly maintain this ‘other (online) life’ in secret for many reasons: To avoid conflicts, to be judged, along with other reasons. 

Concerning the consequences (see Table 9), the positive outcomes are usually associated with enriching users’ social lives, as they could experience other ‘virtual (online) worlds’ and relationships that they consider improving their lives. For instance, they can connect differently with others who are more related to them (i.e., transmitting the online ‘virtual self’ through SNS or through an avatar in a game, creating and maintaining friendships or affective relationships through private online spaces in SNS or through games (e.g., ‘feeling together’), dating and finding partners). They have other types of connections parallel to their ‘real life’ ones (i.e., relatives, partner, peers, colleagues, friends, or other persons with whom they interact face-to-face). Furthermore, gamers report an improvement in their cognitive processes (e.g., memory, attention, and learning). 

However, the adverse self-perceived outcomes by heavy users are mainly those problems which have been unconscious developed until they became aware of the addiction problem, as something (or someone) alerted them. This discovery usually requires them to start behaving differently to naturally recover from a maladaptive to an adaptive pattern of usage. Only in gamers has natural recovery emerged as a process of maturity (i.e., need to develop other life challenges), such as to enter the workplace (which requires responsibility, time, effort, energy, among other skills before invested above all in the game); although, sometimes, they cannot do it by themselves. For instance, it has been reported heavy gamers are living in ‘another world’ which is alternative to real life, and not all of them know how to manage both or do not see the benefit in renouncing ‘virtual life’. A few of them have lost studies/jobs, friends/partners, even some aspects of their health; a few have experienced chronic sleeping and eating problems, and discomfort in some bones, muscles, and organs, probably due to the maintained tension during extended periods (e.g., cervical, hands, or eyes, respectively). Those who mix gaming with drug consumption (e.g., cannabis) experience other problems (e.g., see images of the game when the player was not gaming). 

With regard to neutral consequences, users appeal to curiosity and relationships reinforced by the virtual connections. It is observed that female gamers seem to have boyfriend gamers, as they have met them and/or matched with them better as a couple because of sharing this hobby or passion for games, which ensures their common understanding as gamers and lets them share their virtual lives. Similarly, social networkers report the relevance of being intellectually fed by being updated continuously. Thus, related to these consequences, detail has been provided only for SPIUs. 

Finally, few risk factors were reported and directly connected with the symptomatology in all Internet use-related addiction problems (see Table 10), where developmental and contextual factors seem the most impactful ones. For instance, some of them are: Parent styles, such as authoritarian, partner already ‘addicted’ to gaming, drug consumption together with gaming, the tendency to be indoors with online hobbies, lost someone(s) loved, adolescent crisis, games substituting ‘virtual’ prices with money. Only a few protective factors have also emerged (i.e., family limits, activities outdoors, and transforming the online activity to a healthy activity or profession: ‘eSport’).

#### 3.2.3. Themes Related to IGD Criteria Adapted for the Three Problems

The nine IGD criteria adapted (to excessive general Internet use, video game use (original IGD), or social networks use) were first quantitatively analysed by codes: by frequency (i.e., 2 = essential criterion without a problem detected in its wording; 1 = good criterion with a problem detected; 0 = not an adequate criterion with problems detected; thus, the minimum score was 0, and maximum was 18); with some quotes as illustrations presented in Table 11 below.

The criteria were ranked by order (i.e., from higher to lower frequency of agreement) and the most positively valued and important ones were: Risk or loss of relationships or opportunities, giving up other activities, withdrawal, and continuing despite problems. Following the first set of crucial symptoms which emerged, a second rank order appeared: Escape adverse moods, deceit or cover-up, preoccupation, and being unable to reduce or stop. Moreover, users stated that some of these criteria should not be stated like they are proposed in the criteria set, and in the scales, as usually users are not aware of them and probably select that they do not fulfil these criteria when they do, but are unaware of it (i.e., false negatives). This was usually happening in the following criteria: Preoccupation, reduction or stop, continuing despite problems, deceit or cover-up, and risk or loss of relationships or opportunities. Thus, clinical external evaluation is needed to explore if criteria are met or not by the user. Almost all criteria were well valued except tolerance, as users requested that it be reworded or that it cover not only quantitative time online, but also frequency, money spent on the game, accumulating points/accomplishments/likes, along with other similar elements.

## 4. Discussion

This mixed methods study had as a primary aim to understand potential technological behavioural addictions in young adults based on their self-reported online uses (i.e., Internet, gaming, and social networking) to compare generalised versus specific addiction problems and verify if these could be classed as public health outcomes. This approach has added an innovative research study type into the literature of this field, as the individuals were targeted based on unique trends of Internet use, gaming, and social networking simultaneously in a mixed methods study. The specific aims were: To validate the generalised and specific CIUS to compare these problems, to estimate their prevalence, and to examine problem users’ perceptions regarding the phenomenology of these problems, and their opinion about the IGD criteria for the GPIU and SPIUs studied. 

Concerning the first specific aim, the main psychometric properties were outstanding, showing the commonalities of the three CIUS adaptations tested. Findings showed excellent reliability in all versions (Cronbach alpha coefficients: From α_CIUS_ = 0.90 to α_CIUS–G_ = 0.95), which were even higher than the previous French version [52]. The scales’ factor validity was consistent with its unidimensional model [39], achieving greater explained variance than the original version (from CIUS: 49% to CIUS–G: 60%), and with factor loadings being high enough (from CIUS with a minimum of 0.53 to CIUS–SNS with 0.58). 

Regarding the construct validity, positive associations between the overall scores of each problem and time variables supported the degree to which the adaptations measure what they claim. Nevertheless, differences among the three problems started to appear. For instance, a device profile emerged for the different problems: The GPIU is mainly associated with computer use, although it was also moderately related to time spent per day with other devices (e.g., tablets and smartphones). Gaming only was modestly associated with daily time in computers and tablets, and social networks above all in smartphones (a part of computers on a weak basis). Thus, gaming is not a usual problem when playing on the mobile phone [44,59], as probably modern smartphones and mobile game apps are not yet developed enough for gamers, at least in Europe. However, computers seem to act as an object of these addiction problems and the Internet can provide a vehicle for facilitating some of the addictive symptomatology, as reported in other behavioural addictions (e.g., gambling [60]). 

Concerning content validity, the three forms measured different facets of the ‘Internet addiction’ umbrella construct. For instance, general use was weakly associated with a set of common online behaviours, with messaging, viewing, social networking, and sex consumption standing out. An issue which emerged is the taste for online TV shows/series and film consumption, a recent research line developed in the field [61,62,63], which is usually linked to Internet addiction and gaming as a sedentary behaviour affecting mental health [64,65]. Regarding gaming, game genres with a strong association to this problematic use included by order: MOBA [66,67], MMORPG [50,68,69,70,71], FPS [70,72,73], and solo games [71] (i.e., almost all big games). It is unusual that MOBA and especially ‘solo games’ have emerged, as the literature around problem gaming tends to include only role-playing games. The ‘solo games’ genre makes sense based on the qualitative findings associated with the aetiology about excessively play to avoid or prevent loneliness and boredom, reported in problematic gaming research [71,74,75,76,77]. On the other hand, regarding problem SNS usage, only Facebook emerged as the leading network with which users present addiction symptoms similar to Internet addiction, as previous studies reported [10,78]. As far as the author knows, it has scarcely been clarified what activities are really under the labels of GPIU and these SPIUs, such as gaming [79,80]. 

Interestingly, another set of differences was found between the three problems at a psychometric level. The general Internet and social networking overall scores were strongly positively correlated, which probably means that their constructs are very close, as current SNS have plenty of resources (e.g., Facebook have options to communicate, to share files, to play games, to consume information, even online gambling) that are starting to emulate a generalised online use [10,78,81]. Moreover, the findings of the content validity supported why the general purpose of the Internet and the specific use of social networks were so close in their interpretation. This could explain why when analysing the scales by items, the CIUS and CIUS–SNS have a similar pattern with moderate scores (i.e., CIUS: a *M* from 0.78 in item 9 to 2.05 in item 12 out of 4; CIUS–SNS: An *M* from 0.63 in item 4 to 1.84 in item 1) compared to the CIUS–G (i.e., an *M* from 0.22 in item 9 to 0.66 in item 1). However, the association between general Internet use and gaming was weak, and it did not exist between gaming and social networking, meaning both SPIUs were independent between them. Moreover, qualitative findings support this different phenomenology of both constructs, as users state many differences between problem gaming and problem networking (e.g., the first being detached from real life, while the second overly attached to real life). This independence observed could be interpreted as those who mainly use SNS are not heavy gamers, and inversely [82]. This is a crucial finding as, from a phenomenological perspective, this indicates gaming could not be classed as a social networking problem and vice versa. These findings also suggest that while from an adult’s perspective, their potential addictive symptomatology is higher when using the Internet in general or when networking, only a few users play games in an excessive and problematic form, and these are all types of adults (i.e., males and females, young and middle-aged ones). In a recent study testing the spectrum hypothesis using the network approach, the umbrella construct of Internet addiction was also highlighted; this study recommended the focus on the specific Internet- and technology-mediated addictive behaviours: Gaming, smartphone, and cybersex [83]. Present quantitative and qualitative findings support the specificity of the SPIU of gaming. Regarding gender issues, the differences were detected on SPIUs at a descriptive level, as while descriptively males tend to be gamers and view online porn, females tend to use social networks, as Andreassen and colleagues also reported [82]. However, when analysing only those classed as problem users, findings change. 

Concerning the second specific aim, the estimated prevalence of potential at-risk addiction problem users, findings were (in descending order): 37.4% Internet addicts (generally women and young adults), 32.5% problematic social networkers (usually women and young adults too), and 10.8% problematic online gamers (both genders equally, as well as all group ages in adulthood). Thus, this suggests women are starting to be problem users, especially in addictive gaming, which traditionally was a problem in males [84]. Moreover, while young adulthood has a higher prevalence in general Internet and social networking uses, in the case of gaming, all adults have a similar level of addiction problems, as previously reported [85]. These findings provide evidence for IGD proposal and gaming disorder [6,7], as the problem is extended to both genders and adult group ages. The prevalence estimated was higher than usual for a European study, as reported previously by Laconi and colleagues [80,86]. This overestimation is probably due to a set of methodological factors, the different scales selected in similar studies (e.g., the Internet addiction test; IAT [5] vs. the CIUS [39]), the different interpretation of the symptoms measured as addiction criteria (e.g., see Table 1), and the cut-off score applied to the CIUS, which could be considered a threshold for risk instead of an addiction problem (i.e., as the theoretical median of the CIUS is 28, and 21 was used based on Guertler and colleagues’ study [47], which it was found in the sole self-reported measures from German problem and pathological gamblers, but without clinical support as it is usual in the field). Furthermore, the cut-off was not performed by gender and by type of problem use, and the present sample was mainly composed of women, as it was based on Belgian academic environments of social sciences in higher education. However, almost no research has provided validated cut-off scores using the CIUS, except for the short CIUS [87], which implies a future research line. For this reason, prevalence should be taken with caution as a proposed proportion of users in risk of potential GPIU or SPIU. 

Moreover, as a usual practice in the field, potential problem users were chosen by a psychometric measure, without explicit attention to the symptoms accomplished and their relevance [18,21,22,28] or clinical supervision. Other problems were the results obtained by endorsement per items, as there is no agreement on how to operationalise it (e.g., only extracting those who state ‘often’ and ‘very often’ or only ‘very often’ [88]). Interestingly, a clear difference between core and peripheral symptoms has not clearly emerged by the GPIU and SPIUs studied, contrary to other studies [89], as even the core ones were not related to time spent playing, unlike in previous research [25,26]. It is probably because the quantitative sample contained fewer gamers and problem gamers (the lower prevalence), and usually only players of the ‘big games’ seem to be the ones with more addictive problems [25,26,66,67,68,69,70,72]. Nevertheless, in the qualitative findings, the main addiction problem was gaming through ‘big games’, covering all symptomatology and causing more harm compared to the other two problems studied. 

Thus, only half of the sample could be classed as potential or at risk of being a problem user, a quarter as having a potential Internet use-related addiction problem, and another quarter more than one problem (i.e., mixed profile: Usually by problem general Internet and networking uses), such as in another European study [80], which was closer in its phenomenology, as reported. It is worth noting that most prevalent symptoms in the quantitative evidence on GPIU and SPIUs studied were not those classed as core criteria according to Charlton and Danforth [25] (e.g., conflict, withdrawal) and Tao and colleagues [21] (i.e., preoccupation, withdrawal). Indeed, the commonality between the GPIU and SPIUs studied at a symptomatologic level was with items addressing loss of control and mood modification. This could highlight the fact that potential problematic users are being classed by criteria which are not the core ones, at least in problem gaming; in other words, the overestimation of these potential problem users could be due to a proportion of them being high enthusiasts of using the Internet in general, gaming and/or social networking. In this study, quantitative evidence has highlighted the loss of control was the symptom with high scores, and according to Griffiths, this is not central to addiction [90] (i.e., it is not a necessary component or a consequence of addictive behaviour). However, a few studies reported a difference in the conceptual and nosological entities of these problems using quantitative approaches, especially between GPIU versus SPIU (i.e., Internet addiction vs. problem online gaming [80]), although the distinction was mainly based on gender, which it seems to be changing based on present findings. Furthermore, the female gender seems at risk of these potential behavioural addictions, even gaming, which is a novel finding against the current literature [91]; this highlights that more research in female gamer in behavioural addictions is needed.

Regarding the third specific aim, the aetiology observed through the qualitative evidence shows more causes have emerged than in previous qualitative studies (e.g., usually only focused on boredom, mood feeling, stress, and escapism [58]); for instance, being an indoors person, with an introvert personality, real and virtual friendship with the guild, and being tech-savvy, feeling the need to be updated, connected, and obsessively informed by others in the case of social networkers. Curiously, environmental factors, such as weak or perceived difficult interpersonal context, have emerged as facilitating problematic gaming, but not other PIUs. Thus, maybe not all external factors facilitate same GPIU or SPIU. Similarly, Griffiths’ component model was effective only for problem gaming [18], as a phenomenon caused by a biopsychosocial perspective; followed by problematic social networking; and finally, GPIU with less symptoms, which do not facilitate covering the need for more evidence to promote theoretical development on GPIUs [14] (e.g., only relapse was present as a symptom in problematic gaming). 

According to addiction symptoms, salience seems not to only be a cognitive component, as its emotional facet has emerged, as it was stated by Griffiths [18], and it appears to include craving; however, it was not involved in other Internet addiction criteria or IGD [21,22]. Moreover, this addictive symptom is usually used to diagnose SUDs and gambling disorder, but not included in, for example, IGD [6,92]). Davis’ model [15] did not cover this affective aspect of the nature of GPIU or SPIUs. Indeed, Caplan statements about the preference for online interaction, mood regulation, and deficient self-regulation [15,16] were supported by all interviewees. However, tolerance is more complicated than stated in the most contemporary proposals to diagnose behavioural addictions [21,22], especially in gaming [33]; as it is the increment of not only time (i.e., intensity, expertise, or advancement in the game), and not only for excitement or desire (wording usually used in IGD [22], which has been highly criticised in the interviews). Concerning deception or cover-up, like tolerance, it is more complicated than initially described in addictive symptomatology [18,19,20,21,28,33], as well as in the existing scales (e.g., the IAT [87]). It seems gamers usually maintain a ‘secret double life’ in the game, as they have explained, and as it has scanty been reported as a shared virtual environment among MMORPG players [93,94]. They have ‘game friends’ (the partner, guild or other teams) and their codes to communicate (own language), dedicating daily time to set goals and enjoy within the virtual world of the games; this essential element of gaming behaviour has not been studied in-depth yet [95]. If quantitative and qualitative findings are observed simultaneously, according to Tao and colleagues [21], preoccupation and withdrawal are weakly and moderately present, respectively, in the main sample. However, they are only present in the problematic gamers subsample, and persistent desire and difficult to control are strongly present in the sample for all (GPIU and SPIUs) studied, as well as in all problem users studied. Thus, based on Tao’s criteria, problems studied could be classed as Internet use-related addiction problems with caution; only gaming seems to fit in their proposal. Nevertheless, for IGD [6,22] criteria, only gaming could also be categorised as SPIU based on all findings. Remarkably, the core components [25] could just be checked on the qualitative findings extracted from potential problem users, which concurrently stated conflict was the main addiction symptom (i.e., the one with a higher frequency of agreement), followed by withdrawal, giving up other activities, and continuing despite problems, as probable indicators of behavioural salience. In other words, matching all Charlton and Danforth core criteria for addictive gaming [25,26], qualitative findings support the health problem, together with IGD opinions, which are considered. 

Regarding the consequences of excessive online uses, it should be highlighted not all of them are negative. Almost all of the users agreed they have experienced (or observed in others close to them) the risk of being addicted to the Internet or an online activity (usually gaming). They reported developmental and contextual factors as those supporting how a habit such as gaming could slowly and unconsciously develop into an addictive behaviour. For instance, to be a gamer and play video games during adolescence indoors during a long time, avoiding loneliness, establishing an identity in the game, and virtual social life may relate to real life (e.g., real friends are also gamers of the same role-playing games). The risk factors highlighted compared to the symptomatology reported [18,19,20,21,22,23,24,25], which were connected to both developmental and contextual factors (e.g., adolescent crises, parent styles) were found to be more important than individual factors (e.g., personality traits). This assertion goes against our current research in the field (i.e., focused on personality traits [73,96]), while few contextual or structural risk factors [97] or protective factors have been reported. Nevertheless, it should be argued that maybe these participants are not so aware of their characteristics that may help to develop extreme online behaviours, as in other addictive problems (e.g., SUDs). However, none of the interviewees recognised they might be a problem user at present, which questions the cut-off score selected in the adapted CIUS or supports that they probably had (or have had) an addiction problem (i.e., only one gamer recognised to have had this problem in the past, and another gamer described the game transfer phenomena [98]). 

Finally, with respect to the fourth specific aim about IGD criteria opinions for diagnosing these problems, qualitative findings highlighted as the most critical addiction symptoms: Risk or loss of relationships or opportunities (conflict), giving up other activities, withdrawal, and continuing despite problems, which coin the core components previously pointed out [25] and match research findings [33]. Moreover, users clearly state ‘intrapersonal or interpersonal conflict’ [18] is equally important, sometimes associated with a ‘functional impairment’ [21]. The qualitative evidence seems to agree with critiques considering GPIU with not enough entity to be as a behavioural addiction [23,80], as a few subthemes did not provide evidence (e.g., not emerging in all addiction symptomatology analysed). In this sense, other excessive online behaviours, such as watching series online, have emerged and caused public health concerns with sedentary lifestyle and time in front of the screens [61,62,63]. Other criteria, such as escaping adverse moods, deceit or cover-up, preoccupation or not being able to reduce or stop online behaviour, are quite ordinary and excessive users agreed with them, the only criterion where users disagree being tolerance, one of which Kardefelt-Winther [24] criticised in the current IGD [22], and about which other authors have found similar evidence [33]. Tolerance, giving up other activities, or escaping adverse moods require a part of rewording them and covering their complexity, an indicator of frequency, as Ko [99] also stated when IGD was published in the DSM-5 appendix [6], as the need for using the intensity and frequency criteria to distinguish subjects with IGD from casual online gamers.

Some limitations of this cross-sectional and self-report study have been already discussed, but it could be added that it was performed with a non-random community and academic sample, with an online survey and interviewing only those who accepted the invitation to participate in the second part of this study (e.g., economic motivation). However, similar quantitative studies have been performed with smaller samples than the quantitative [80] and qualitative [50] parts of the study. The study’s strengths include a survey with psychometric and epidemiological validated techniques, with a considerable and sufficient sample size based on Nunnally’s recommendations for psychometrics [100], and Smith and Osborn’s assertion [101] for the qualitative part of the study. The provision of a Belgian French adaptation of the CIUS and IGD for specific Internet use-related addiction problems was supported by previously validated adaptations [22,52]. The qualitative participants were psychometrically and theoretically selected after validating the CIUS scales to have the potential problem online users from all profiles studied to shed light on the phenomenology of these new addiction problems from adults. 

In summary, technology uses are growing within contemporary societies and bibliographic productivity is attending these phenomena quantitatively. However, confirmatory approaches could have been chosen as a method for factor validity, which is a future research study under development. Finally, although this is not the first mixed methods study done in the field [33,92], it is one of the few that have been undertaken covering mixed user profiles of these problems. Therefore, it is an original contribution looking for mixed evidence on what seems to constitute the phenomenology of these problems through potential or at-risk adult problem users, although no functional impairment or other possible mental disorders were measured to disentangle the problem users with a clinical approach. 

## 5. Conclusions 

The present study is one of the first to ascertain what seems to be behind current psychometric measures and to start to disentangle generalised and specific Internet addiction problems, addressing their phenomenology with quantitative and qualitative evidence. 

The findings show that around half of Belgian (European) adults seem to present at least a risk of Internet use-related addiction problems. However, a lower proportion could have a mixed profile, covering the risk of more than one problem. The CIUS for generalised problematic internet use and specific problematic gaming and social networking has been validated, and the potential prevalence in at-risk users have been estimated. Young or middle-aged adults in both genders have been shown to be equally vulnerable to problematic gaming. Furthermore, the addiction symptoms are present in the problems studied, but not with the same weight or level of importance. The higher scores are in general Internet addiction and problem social networking, which seem to have a common phenomenology, while problem gaming seems different. However, all of them have loss of control and mood modification as main prevalent symptoms. Nevertheless, when problem gaming is analysed in-depth, it is difficult to confirm all addictive components, symptoms, and criteria, in quantitative and qualitative findings. Furthermore, the classic core components have been tested but were only ratified in the qualitative results, where the relevant symptoms were conflicting, giving up other activities, withdrawal, and continuing despite problems. 

An in-depth theoretical and empirical review of the public health approach usually used in behavioural addictions literature applied to generalised and specific addiction problems has been provided. This strategy together with analysing quantitative and qualitative evidence of these problems has shown what seems to be behind each addictive criterion usually used to screen users and classify them as a potential problem user (i.e., through DSM criteria, component addiction symptoms, and core vs. peripheral symptoms). Moreover, it has been documented that at-risk users’ perceptions about the phenomenology of these problems, which start in adolescence and remain during adulthood, with a potential of increased harm. The aetiology is more diverse than previously described, and its development is supported by psychosocial, environmental, and technological factors, leading to a few cases of extreme problems. Potential at-risk problem users’ opinions about IGD criteria have highlighted that some criteria (e.g., risk or loss of relationships or opportunities) are more important and better developed than others (e.g., tolerance), but the need to apply them with clinical resources is essential in the evaluation and diagnosis of these mental health problems. Nevertheless, natural recovery has emerged as a finding for some potential problem users, although mental health systems to support those users who have developed the problem(s) and cannot naturally recover are required. Other preventive actions in education, social, and family relationships could promote the maintenance of users’ health and wellbeing. 

Thus, it seems these problems exist, but probably in a lower proportion than reported. However, when they do appear they present addictive symptomatology, especially in problem online gaming. These findings seem to provide evidence for the IGD proposed by the APA (with the restrictions and improvements suggested) and the recent recognition of gaming disorder by the WHO (especially for specific online gaming behaviours). 

## Figures and Tables

**Table 2:** Descriptive, reliability, and correlation matrix across all adaptations for the CIUS.

		CIUS Adaptations			
Scale Adaptations	Descriptive *M*(*SD*); Range	Cronbach alpha	CIUS	CIUS–G	CIUS–SNS
**CIUS**	14.63(10.10); 0–56	0.90	-		
**CIUS–G**	4.78(8.97); 0–51	0.95	0.22 **	-	
**CIUS–SNS**	11.63(10.79); 0–56	0.93	0.59 **	−0.02	-

Note: CIUS: Compulsive Internet use scale; CIUS–G: CIUS for gaming; CIUS–SNS: CIUS for social networking sites; *M*: Mean; *SD*: Standard deviation; ** *p* < 0.01.

**Table 3:** Item analysis: descriptive, correlation item per total correlation, and factor loadings across all adaptations for the CIUS.

Items	Descriptive *M*(*SD*)			Correlation Item-Total Correlation Per CIUS Adaptations			Factor Loading Per CIUS Adaptations		
	CIUS	CIUS–G	CIUS–SNS	CIUS	CIUS–G	CIUS–SNS	CIUS	CIUS–G	CIUS–SNS
**1**	1.98(1.267)	0.66(1.159)	1.84(1.372)	0.60	0.79	0.71	0.67	0.82	0.76
**2**	1.84(1.354)	0.52(1045)	1.73(1.468)	0.65	0.76	0.74	0.71	0.79	0.79
**3**	0.80(1.089)	0.27(0.765)	0.78(1.131)	0.46	0.63	0.61	0.53	0.69	0.67
**4**	0.90(1.003)	0.31(0.768)	0.63(0.945)	0.56	0.75	0.52	0.63	0.78	0.58
**5**	1.07(1.147)	0.28(0.750)	0.79(1.094)	0.57	0.68	0.64	0.64	0.73	0.69
**6**	0.99(1.045)	0.29(0.716)	1.06(1.087)	0.56	0.74	0.67	0.63	0.79	0.72
**7**	1.16(1.107)	0.39(0.863)	1.00(1.103)	0.56	0.80	0.64	0.64	0.84	0.70
**8**	1.33(1.187)	0.39(0.872)	1.39(1.321)	0.59	0.75	0.65	0.66	0.79	0.71
**9**	0.78(1.017)	0.22(0.615)	0.82(1.094)	0.63	0.64	0.62	0.70	0.69	0.68
**10**	1.01(1.090)	0.29(0.739)	0.84(1.107)	0.66	0.78	0.69	0.72	0.83	0.75
**11**	1.07(1.175)	0.28(0.718)	0.89(1.137)	0.63	0.74	0.64	0.70	0.79	0.69
**12**	2.05(1.240)	0.57(1.095)	1.69(1.419)	0.60	0.79	0.71	0.67	0.82	0.75
**13**	1.54(1.304)	0.41(0.943)	1.18(1.318)	0.59	0.72	0.65	0.66	0.76	0.71
**14**	1.20(1.172)	0.23(0.657)	0.91(1.132)	0.51	0.69	0.67	0.85	0.74	0.73

Note: CIUS: Compulsive Internet use scale; CIUS–G: CIUS for gaming; CIUS–SNS: CIUS for social networking sites; *M*: Mean; *SD*: Standard deviation.

**Table 4:** Descriptive and correlation across all validated adaptations for the CIUS in relation to time.

Scales and Descriptive	Days Per Week (Weekly Frequency) *r*			Minutes Per Day in a Weekly Day *r*			Minutes Per Day in a Weekend Day *r*		
	Computers	Tablets	Phones	Computers	Tablets	Phones	Computers	Tablets	Phones
**CIUS**	0.18 **	0.08	0.09	0.37 **	0.25 **	0.24 **	0.39 **	0.28 **	0.21 **
**CIUS–G**	0.14 **	0.2 7 **	0.07	0.27 **	0.26 **	0.05	0.22 **	0.21 **	0.02
**CIUS–SNS**	0.09	−0.02	0.21 **	0.11 *	0.08	0.35 **	0.22 **	0.13	0.37 *
***M*(*SD*)**	6.17(1.62)	3.87(2.80)	4.62(2.98)	118.71(93.45)	46.10(64.99)	82.70(116.08)	183.91(200.53)	68.16(100.13)	90.99(124.45)

Note: Weekly Day: from Monday to Friday; Weekend Day: from Saturday to Sunday; CIUS: Compulsive Internet use scale; CIUS–G: CIUS for gaming; CIUS–SNS: CIUS for social networking sites; *r*: r Pearson (correlation); *M*: Mean; *SD*: Standard deviation; * *p* < 0.05; ** *p* < 0.01.

**Table 5:** Item and symptoms from potential problem users: descriptive, and frequency of endorsement across all adaptations for the CIUS.

Item Number, Symptom According to Meerkerk et al., 2009 [39] and Charlton & Danforth, 2007 [25]	Descriptive *M*(*SD*)			Endorsement *f* i (%)		
	CIUS	CIUS–G	CIUS–SNS	CIUS (*n* = 176)	CIUS–G (*n* = 51)	CIUS–SNS (*n* = 153)
**1—Loss of control–Peripheral**	2.86(0.942)	2.86(1.02)	3.08(0.924)	114(64.8)	34(66.7)	112(73.2)
**2—Loss of control–Peripheral**	2.91(0.987)	2.59(1.134)	3.07(0.926)	124(70.5)	28(54.9)	113(73.9)
**3—Conflict–Core**	1.48(1.214)	1.55(1.301)	1.73(1.262)	31(17.6)	12(23.5)	38(24.8)
**4—Preoccupation–Core**	1.53(1.008)	1.82(0.974)	1.25(1.079)	26(14.8)	11(21.6)	18(11.8)
**5—Loss of control–Core**	1.85(1.162)	1.59(1.236)	1.67(1.261)	43(24.4)	13(25.5)	41(26.8)
**6—Preoccupation–Peripheral**	1.70(1.039)	1.71(1.082)	2.04(0.973)	39(22.2)	9(17.6)	46(30.1)
**7—Preoccupation–Peripheral**	1.9(1.057)	2.18(1.072)	1.94(1.04)	52(29.5)	19(37.3)	41(26.8)
**8—Conflict–Core**	2.19(1.082)	2.02(0.99)	2.46(1.088)	68(38.6)	14(27.5)	77(50.3)
**9—Loss of control–Peripheral**	1.56(1.057)	1.20(1.077)	1.70(1.181)	29(16.5)	7(13.7)	37(24.2)
**10—Conflict–Core**	1.86(1.073)	1.80(1.114)	1.85(1.146)	52(29.5)	13(25.5)	43(28.1)
**11—Conflict–Core**	1.97(1.146)	1.67(1.089)	1.82(1.227)	53(30.1)	12(23.5)	40(26.1)
**12—Mood modification–Peripheral**	2.92(0.977)	2.59(1.268)	2.95(1.022)	124(70.5)	30(58.8)	113(73.9)
**13—Mood modification–Peripheral**	2.48(1.181)	2.12(1.291)	2.30(1.22)	97(55.1)	20(39.2)	67(43.8)
**14—Withdrawal–Core**	1.89(1.175)	1.43(1.171)	1.90(1.134)	55(31.3)	11(21.6)	47(30.7)

Note: CIUS: Compulsive Internet use scale; CIUS–G: CIUS for gaming; CIUS–SNS: CIUS for social networking sites; *M*: Mean; *SD*: Standard deviation; *f* i: frequency; % is valid percentage; *n*: Subsample size; and item 5 was moved from ‘Loss of control’ [39] to Conflict by the author, for this reason has been classed as Core symptom [25].

**Table 6:** Participant sociodemographic characteristics and potential problem online uses.

Pseudonyms	Variables					
	Gender	Age	Civil Status	CIUS	CIUS–G	CIUS–SNS
Leia	Female	31	Partner	**24**	**29**	17
Moira	Female	20	Single	**26**	5	**28**
Aneka	Female	20	Partner	**37**	14	**37**
Victor	Male	20	Single	**36**	**31**	**37**
Elektra	Female	35	Divorced	**23**	5	8
Carol	Female	21	Partner	13	**25**	19
Scarlet	Female	18	Single	8	0	**21**
Martin	Male	19	Partner	9	**22**	4

Note: CIUS: Compulsive Internet use scale; CIUS–G: CIUS for Gaming; CIUS–SNS: CIUS for Social networking sites; numbers in bold are the scores above the cut-off in each CIUS adaptation as potential problems.

**Table 7:** Superordinate and subordinate themes about the aetiology of the SPIUs studied, together with a few quotations (adapted from French to English) as illustration.

Theme	Subthemes		
		CIUS–G	CIUS–SNS
Causes	Individual facet	‘I started to be a gamer at the same time I started at the university. It is quite usual in people who like technologies to have online hobbies, as we have difficulties communicating with other people; games could be an escape’ (Leia) ‘I think the pathology in gaming appears when you cannot avoid craving; when the negative feelings emerge from not being able to play, it is the lack of something in you’ (Victor)	‘In the game, when you win you feel you are valorised; on Facebook, you only see positive things and this makes people feel positive, as it is easier to see someone through Facebook than call him or her’ (Elektra)
	Social facet	‘What makes me play more and more is playing online with those I know, to compete among us’ (Victor) ‘I play League of Legends, a MMORPG, not a MOBA, as it needs an objective and it is a network; you play with friends, and this is what I really like’ (Martin)	‘It is the wish to share; as sharing your emotions. Above all when there is good news you share it through Facebook’ (Moira)
	Contextual facet	‘Gaming can increase if there is a lot of free time, studying at university or being unemployed. Also because external relations are difficult, at school, friendships and above all in the family’ (Leia)	‘With all technologies around us, you feel the obligation to be connected all the time, to know what is happening’ (Moira)

Note: CIUS–G: Compulsive Internet use scale for gaming; CIUS–SNS: CIUS for social networking sites; MMORPG: massively multiplayer online role-playing games; MOBA: multiplayer online battle arena.

**Table 8:** Superordinate and subordinate themes about the development (i.e., addictive symptomatology) of the GPIU and SPIUs studied, together with a few quotations (adapted from French to English) as illustration.

Theme	Subthemes	Quotes		
		CIUS	CIUS–G	CIUS–SNS
Development (addictive symptoms)	Salience	‘If you have a need, such as looking for a job, and you always have your smartphone with you, you could get obsessive about checking the Internet continuously. You are in your own world. If I am not checking it, I think I am losing opportunities’ (Aneka)	‘Gaming is excessive when it is a priority. Years ago, I felt I needed to play when I was going out with others, and I realised I had a problem. It is not a question of time playing. It is about when you cannot be without gaming, and there is no Internet. For instance, a gamer will take public transport for hours until finding a place with Internet to play video games. The gamer substitutes going out with friends for video gaming, and gamers are always connected even through a smartphone’ (Carol) ‘There are guild obligations, as we agree to be there to do something, and if you do not do it, you put people in trouble’ (Leia) ‘The ‘vagaries’ of life, there are periods when I wish to play. I do not think craving is in myself or my brain; I think it is outside’ (Carol)	‘In the SNS we think of other things, to free our minds of negative feelings’ (Moira)
	Mood modification	‘If I am sad, I watch an online series or films to cheer up, which sometimes is better than gaming’ (Carol)	‘I integrate myself in the story and into a character of an MMORPG to disconnect with reality. When you have had a stressed day, your reward is gaming. As I made an effort, I have the right to escape; it is very relaxing’ (Carol) ‘When gaming (RPG or FPS) we can quickly become annoyed or nervous, but sometimes it’s the contrary, playing casual games helps you to relax. I have all types of different games I play depending on my mood, and how I want to balance my emotions’ (Leia) ‘When I am alone and upset, I play to calm myself, but I do not regulate myself as I feel tense or nervous after stopping’ (Martin)	‘You go to Facebook to look for something to cheer you up’ (Moira)
	Tolerance		‘The feelings of success and gratification could be a stimulus that makes us think of the game and makes us feel well, and produces the wish to play’ (Carol) ‘I need to have my little dose every day to feel like I’m advancing in the game. Before I could play for 15 hours daily, but now only 2 h. For example, if you stop for a day everything is reset, so sometimes we have to play to keep the game and the gamers together, and when you win you are happy’ (Leia)	‘It is the need to look into other’s lives. It is about being jealous, to posting pictures, messages, and to observing reactions through the numbers of likes and comments we receive, as these reinforce you’ (Elektra)
	Withdrawal		‘Ten years ago, I had to play games on my computer. If I couldn’t, I was frustrated; it was emotionally automatic. I had only one desire: to enter the game and play’ (Leia) ’Once we were on vacation for weeks and I could not access the Internet, I felt the craving. I was thinking all the time about it. I learnt I could not put gaming as a priority, and I started to control the periods of gaming or not gaming’ (Carol)	
	Conflict (intrapersonal and interpersonal)	‘Problematic use is when the use of the Internet is affecting the family or the couple. Or if it is affecting sleep, work, or social life’ (Elektra)	‘I was gaming in class sessions, when I came back home I spent whole nights on my computer gaming. I could play 15 or 16 hours per day, but other people left or hindered their studies or jobs, or had conflicts with their partners’ (Leia) ‘You neglect other activities, your course, your family, other social contacts, even your health. Gamers have a lack of vitamin D because they do not go out, they eat poorly and quickly, so they can return to the game’ (Carol) ‘Excessive gaming is evident as lack of sleep shows in your eyes; hygiene, as you are not taking showers for gaming; the body, as you skip meals. These have consequences for your family, life, and work. It is the same problem during adolescence or adulthood, but the consequences are worst in adulthood. It causes tiredness. It is a loop’ (Martin) ‘I think the more you play, the more it affects your vision; as you start to seeing images from your thoughts; and these are engaged with the game’ (Martin)	‘When I am online too much, without sleeping and with troubles in my daily life, I have observed others like me have real problems with their studies, with both games and SNS’ (Moira)
	Relapse		‘If you want to leave a game such as a FPS, the group require you to return to maintain the same number of gamers in the teams as before. If not, they need to look for other gamers who are not so good. It is like a team sport, we have microphones, it is not simply a game, and it is another dimension. It is about speaking, planning strategies, indeed it is a world that we develop for a long time. Thus, when dates are fixed it is too restrictive’ (Victor)	
	Difficulty to control it	‘When I come home, I connect myself to Facebook from 5 to 10 or 11 pm, and if I cannot sleep, I use the Internet’ (Aneka)	‘Each new version of an RPG causes an increment of gamers and game play again. These are persistent worlds and being in one country or another does not change anything’ (Leia) ‘You make a false plan; it’s almost a fake virtual plan’ (Victor) ‘One of my relatives was online gaming in an RPG with a guild, and we told him he needed to do something else, but he did not stop’ (Victor)	‘When I play with my partner, he cannot stop gaming if he is not winning. It is a fact of being successful, to have a goal. When a gamer develops an addiction, I think it is because they are attached to a world which is not real life. This is different from SNS users who are overly connected with real life. Gaming and SNS are really different’ (Moira)
	Continued use disregard		‘I passed hours gaming even when my eyes hurt’ (Martin)	
	Deceive		‘I had an alternative life; it is like private groups in Facebook, but in the game, and we are constantly in communication through software. We have the impression of living a double life, we do not speak about our real lives in the game, because people ask questions and judge us. It is a secret double life, it is a habit, like a drug’ (Leia)	

Note: CIUS: Compulsive Internet use scale; CIUS–G: CIUS for gaming; CIUS–SNS: CIUS for social networking sites; MMORPG: massively multiplayer online role-playing games; RPG: role-playing games; FPS: first-person shooter.

**Table 9:** Superordinate and subordinate themes about the consequences of the GPIU and SPIUs studied, together with a few quotations (adapted from French to English) as illustration.

Theme	Subthemes		
		CIUS–G	CIUS–SNS
Consequences	Positive	‘I play for the strategy, the research, the challenge, the learning through connections of things. We get into to a story and this promotes your memory, intelligence, and the capacity to maintain attention and quickly answer, to plan and foresee consequences, to adapt yourself. We are less confined in a virtual world and we could see and live more and differently than in real life’ (Leia)	‘Facebook could be a place to meet with my partner, to communicate or play games. I need to maintain this bond daily to maintain our news’ (Moira)
	Negative	‘Gaming is addictive without knowing exactly why. The people around you or your financial situation will not stop you, only the circumstances such as the professional world, physical problems, and a partner if he or she is not a gamer. Gamers can be confined and isolated; some of them have lost courses, jobs, partners; it can be dramatic’ (Leia) ‘Online games sometimes cause negative consequences at a social level, because these are simultaneously promoting isolation of the gamer; maybe there were previous social problems which promoted this isolation through online games. In any case, there is a progressive reclusion, I have observed this in a close friend’ (Victor)	‘Playing games excessively is a step out of reality; they should go outdoors more, as SNS users usually do’ (Moira)
	Neutral	‘Gaming is for curiosity, for enjoyment; gaming makes you happy; it does not always affect your real life’ (Carol)	‘I am also very curious about newsfeeds’ (Moira)

Note: CIUS–G: Compulsive Internet use scale for gaming; CIUS–SNS: CIUS for social networking sites.

**Table 10:** Superordinate and subordinate themes about the prevention of the GPIU and SPIUs studied, together with a few quotations (adapted from French to English) as illustration.

Theme	Subthemes	Quotes		
		CIUS	CIUS–G	CIUS–SNS
Prevention	Risk factors	‘A close friend lost a relative, and he was gaming between 5-6 hours per day, plus watching online series, which affected his studies. It was to compensate for the loss. Now he has reduced his gaming and we do other things’ (Carol)	‘I had a growing crisis in my adolescence; I had to detach from my family, and I could not do it physically, only through the games. I discovered another world and friends’ (Carol) ‘When I was an adolescent, I spent a lot of time at home alone gaming. In University you live alone, and you do what you want’ (Martin) ‘I had a friend who had difficulties with his father, then he played games to prove his value, as he needed to valorise himself, to obtain recognition’ (Carol) ‘I had a boyfriend who introduced me to gaming; it became a vicious circle. There were a lot of external thoughts that made us think about the game, remembering our wellbeing when gaming or the feeling to do something which challenged us was hard; but I started to look for real accomplishments’ (Carol) ‘I know someone who was an extreme gamer, and he smoked cannabis and played video games simultaneously which became a habit. The cannabis was only reinforcing the habit. The cause was that he was living alone with a parent who worked a lot. He only stopped to help his parent, but he spent the money to buy things for the MMORPG, or to buy cannabis’ (Martin) ‘There is a system that makes you play more, as you win and you are repaid receiving points. These points let you buy characters without real money; although you could buy things for the game with real money. It is a vicious cycle. The virtual money is the points you accumulate, and there is a ranking; then reputation also makes you play more’ (Martin)	‘A trauma could encourage you to stay behind the computer’ (Moira) ‘I had a close friend who was using the SNS all day until she found a partner. Problematic use could appear in a period of solitude to replace the lack of relationships with others’ (Elektra) ‘I was on Facebook a lot when I broke up with my partner; to avoid thinking’ (Scarlet)
	Protective factors		‘I had a friend who could not control his time online. I recommended dancing to him, as I had other hobbies apart of the Internet, like dancing 4 hours per week which diminished the hours of my gaming’ (Carol) ‘There is eSport at a global level, which is a way to win money, where you have to have real teams to compete. They are famous, and win a lot of money with sponsors. I think these gamers are not addicts, as their rewards are real, they are professional gamers; this is a career’ (Martin)	‘My parents were against the technologies. We did not receive education about them, and we were too connected at home. Thus, they started to switch off the Wi-Fi in the evening, encouraged us to go out, to start doing other activities: dance, music’ (Moira)

Note: CIUS: Compulsive Internet use scale; CIUS–G: CIUS for gaming; CIUS–SNS: CIUS for social networking sites; MMORPG: massively multiplayer online role-playing games.

**Table 11:** Internet, gaming, and social networking criteria encoded through the frequency of agreement and a few quotations (adapted from French to English) as illustration.

Criteria	Frequency of Agreement	Quotes
1: Preoccupation	∑ = 8	‘This is a good criterion, if we are only thinking about what is happening through Facebook, it’s as if we are addicted to it’ (Moira, as a social networker) ‘It remains in your head, we are permanently thinking, but it is also constantly in your feelings. Thus, I think this criterion should include feelings, and the cause of the suffering’ (Victor, as a gamer) ‘I would eliminate the phrase ‘a lot of time’, as it requires reflection’ (Elektra, as a social networker) ‘The problem is when you recognise your strange behaviour when not gaming: the gamer is thinking of the game outside the game, such as reflecting on strategies for playing. I have also observed this in girls who excessively shop online’ (Carol, as a gamer)
2: Withdrawal	∑ = 10	‘This is a good criterion, it is difficult to stop using Facebook. I am a bit addicted as I am on it 3-4 hours a day. I have started to tell myself I should do another thing in these hours, but I have not stopped. It is a need, but it is different from other online activities because it’s about being in touch’ (Moira, as a social networker) ‘This is the most important criterion because the diagnostic includes suffering, what the user feels’ (Victor, as a gamer) ‘I think it should be divided into two criterions as there are two different verbs which refer to two different things’ (Elektra, as a social networker) ‘These excessive gamers can be with you to a degree, but they are so nervous and anxious to return to the games’ (Martin, as a gamer)
3: Tolerance	∑ = 1	‘Only if the game is not on computers. I think it is not that relevant, as I did not have this need as stated, but I was addicted to games’ (Leia, as a gamer) ‘To have a hobby which you would like to do more or improve is not a problem, as long as there are no financial problems. Everybody is online’ (Victor, as a gamer) ‘I would eliminate the words ‘excitation’ and ‘pleasure’ as these are intimate words, the word ‘satisfaction’ is better. Should add the notion of frequency, as in the SNS we are not spending much time but a lot of times’ (Elektra, as a social networker) ‘When one is addicted to a game there is no need to play more for the same state of excitement; the problem is that we do not want to leave the game. This behaviour is not like drugs; we do not need to increment the dose, above all for MMORPG. For those who have problems, to invest money in improving their materials to play could be a sign. If the gamer has this problem is because he or she has trouble with the excitement. Gaming addicts want to acquire more and more, as the feeling is not to wait, because the game continues without the gamer. I only accumulate accomplishments for avoiding the fear of losing, for not losing events’ (Carol, as a gamer) ‘To have new materials improves your quality of playing games, then more time and more things, the question is complex’ (Martin, as a gamer)
4: Reduce or stop	∑ = 7	‘Some gamers are not aware of their problem, but when they realise, they have already achieved a step towards recovery’ (Leia, as a gamer) ‘I think this criterion is above all for those who spend too much time alone’ (Moira, as a social networker) ‘I think it is for users who are aware of their excessive use, but there are periods in life you are not aware of it; it is a global unhappiness’ (Elektra, as a social networker) ‘You never take the pauses even when thinking of them’ (Martin, as a gamer)
5: Give up other activities	∑ = 10	‘This is a good criterion, but it is important to be aware this affects real life’ (Leia, as a gamer) ‘This is a fundamental criterion. Others stop their activities to be in the SNS’ (Moira, as a social networker) ‘The problem is users could consider their online activity as a sole hobby’ (Elektra, as a social networker) ‘I know I had an excessive gaming behaviour, playing a lot with craving, but nobody knew about it, as I was doing ‘normal’ life in school, and with other activities. You could be an addict and not accomplish this criterion’ (Carol, as a gamer) ‘This is a discriminative criterion, the impact on your life, when will everything be affected. I have lived it. It lacks the notion of time, the frequency when gaming’ (Martin, as a gamer)
6: Continue despite problems	∑ = 10	‘It is a good criterion if SNS are affecting our relationships negatively; as when you are going out to dinner, and everybody is always on their smartphones chatting by the SNS instead of with those who are at the dinner’ (Moira, as a social networker) ‘It is important, but maybe the user is not aware and continues gaming’ (Victor, as a gamer) ‘As the previous one, you could be an addict gamer and not accomplish this criterion, as others will not observe it’ (Carol, as a gamer) ‘This is only useful if the person is conscious’ (Martin, as a gamer)
7: Deceive or cover-up	∑ = 9	‘This is a good criterion. Gamers are a bit ashamed as we live in persistent worlds and we communicate with friends who are gamers’ (Leia, as a gamer) ‘This is a good criterion, if the user is conscious he or she is addicted to SNS’ (Moira, as a social networker) ‘This depends on the context, such as a strict family which puts pressure on you, maybe you will secretly game ‘(Victor, as a gamer) ‘In the moment the gamer lies he or she is conscious’ (Martin, as a gamer)
8: Escape adverse moods	∑ = 9	‘This is a good criterion, as there are users who use the games as a shelter’ (Leia, as a gamer) ‘This is a criterion for those who play video games, but it should differentiate those who play for coping with a traumatic experience and those who play without any excuse’ (Moira, as a social networker) ‘Some gamers play to compensate, but others for the adrenaline, some to be alone, some to be happy’ (Carol, as a gamer) ‘It is needs to include the notion of frequency to achieve the concept of habit’ (Martin, as a gamer)
9: Risk or loss of relationships or opportunities	∑ = 13	‘This is a good criterion too, as it is influencing real-life’ (Leia, as a gamer) ‘This is a good criterion, it is too extreme but exists’ (Moira, as a social networker) ‘It is a subjective criterion because maybe the gamer treats this activity as work and he is fine like this, but if he is not aware and it affects him negatively, it is a problem (Victor, as a gamer) ‘If you say yes, you could be an addict gamer; but if you say no, maybe you are accomplishing the criterion without being aware of it, and you could pass as healthy when you are not’ (Carol, as a gamer) ‘This is the most discriminative, as we lost something for playing and the addiction takes something from you’ (Martin, as a gamer)

Note: ∑ means to sum all quantitative values (codes). SNS: social networking sites; MMORPG: massively multiplayer online role-playing games.
